# Effects of Poly‐L‐Lactic Acid Fillers on Inflammatory Response and Collagen Synthesis in Different Animal Models

**DOI:** 10.1111/jocd.70000

**Published:** 2025-02-05

**Authors:** Tong He, Zilin Zhang, Xiaoting Zhang, Huanyun Niu, Shiwei Wang, Qiaoli Wang, Chen Lai

**Affiliations:** ^1^ Bejing Engineering Lab of Neo‐Biodegradable Materials Beijing China; ^2^ Biomedical Engineering Center PKU‐HKUST ShenZhen‐HongKong Institution Shenzhen People's Republic of China

**Keywords:** animal model, collagen, inflammation, poly‐L‐lactic acid

## Abstract

**Background:**

The responses to poly‐L‐lactic acid (PLLA) filler implantation exhibit variability across various animal models. This study aimed to investigate these differences in order to identify suitable animal models for diverse experimental applications.

**Methods:**

PLLA fillers were implanted into two distinct skin layers (periosteum and subcutaneous tissue), in rabbits, guinea pigs, rats, and mice. At 1, 2, 4, and 12 weeks postimplantation, biopsy samples were collected for hematoxylin and eosin staining, Masson's trichrome staining, and enzyme‐linked immunosorbent assay (ELISA) to evaluate the differential responses among the animals.

**Results:**

All four animal models exhibited significant inflammatory responses and collagen regeneration following PLLA injection. However, the magnitude of these responses varied across species, with guinea pigs and rabbits displaying the most pronounced inflammatory responses, whereas mice exhibited the mildest. The degree of response also differed between tissue layers, with rats and mice showing stronger inflammatory responses in the periosteum compared to the subcutaneous tissue; a phenomenon not observed in rabbits and guinea pigs. ELISA analysis revealed upregulated TNF‐α, IL‐12, and TGF‐β expression in rats and mice at 12 weeks.

**Conclusions:**

Varying levels of tissue responses are observed following PLLA injections in different animals and within different tissue layers within a single animal. These findings suggest a careful selection of appropriate animal models is necessary for specific research objectives.

## 1 Introduction

The rising demand for esthetic enhancement has led to a widespread adoption of injectable cosmetics for facial rejuvenation and remodeling [1, 2]. This nonsurgical approach effectively addresses subcutaneous volume loss, diminishes wrinkles, and enhances skin texture [1, 3]. A variety of materials have been developed for use as soft tissue fillers, including autologous fat, acellular matrix, hyaluronic acid (HA), and collagen [4]. Moreover, synthetic polymer fillers, such as poly‐L‐lactic acid (PLLA) [5], polycaprolactone (PCL) [6], and polymethyl methacrylate (PMMA) [7, 8], have been created to stimulate collagen regeneration. Notably, PLLA has emerged as the most prevalent collagen‐regenerating filler, boasting a robust safety profile and a long‐standing history of clinical use exceeding two decades [9]. Preclinical investigations, often utilizing animal models, are crucial for validating the safety and efficacy of medical products and elucidating their mechanisms of action [10, 11, 12, 13]. Although the outcomes of these studies are generally consistent, discrepancies in the magnitude and timing of inflammatory response at the injection site persist [12, 14].

For instance, Cabral et al. reported in a study involving Wistar rats that PLLA filler implantation induced a histologically significant response, accompanied by a robust inflammatory reaction, primarily characterized by the infiltration of multinucleated cells, lymphocytes, and macrophages, particularly at 60 days postimplantation [14]. Similarly, Kim et al. employed rats as an in vivo model for PLLA filler implantation experiments in mice, observing multinucleated cells and macrophages, along with a significant granulomatous response and neovascularization [15]. In contrast, Sun et al. utilized rabbits as experimental subjects [16], revealing a more pronounced early inflammatory response and substantial collagen production in response to PLLA implantation compared to the rat models. Dong et al. observed that in rats, the inflammatory response reached its peak at week 4 and then diminished at week 12 following PLLA implantation [12]. Su et al.'s study indicated that the inflammatory response level was highest at week 12 postimplantation, with collagen production gradually increasing over time [17]. In mice, Kwon et al. found that PLLA fillers facilitated collagen regeneration and induced inflammatory responses [18], though the intensity of the response appeared less pronounced in mice. Collectively, these animal experiments demonstrated apparent foreign body reactions; however, inconsistencies in observations across different models were noted. Although variations in PLLA material composition might account for discrepancies in inflammatory response, the inherent differences among animal models also contribute to the observed variations, potentially compromising the objectivity of the findings.

Furthermore, the depth of implantation can elicit diverse tissue responses [19]. Milhomem et al. observed that in mice, the inflammatory response level was influenced by the depth of microsphere implantation, with inflammation decreasing as the depth increased [19]. In clinical practice, fillers are typically implanted in various skin layers, from the middle dermis to the subcutaneous layer, depending on their intended purpose [20]. Superficial placement of PLLA fillers for facial augmentation may lead to more adverse reactions, such as nodules and granulomas, compared to deeper placements [21]. Different skin layers possess varying physiological environments and exhibit differential tissue responses to implants [22, 23]. It is evident that the examination of various implantation tissue layers is essential for studying of material properties and their biological reaction; however, there is a lack of explicit comparative studies to elucidate the impact of different layers in the preclinical evaluation of fillers.

Therefore, in this study, we administered identical PLLA microsphere fillers into different animal models to investigate the divergent tissue responses to subcutaneous injection. Focusing on two commonly used skin layers, the periosteum and subcutaneous tissue, we analyzed the disparities in tissue responses at various injection depths. This aimed to explore the tissue responses of the same filler in different animal models and at different implantation levels, and to provide an initial exploration of common approaches, in hopes of offering a reference for future research.

## 2 Materials and Methods

### 2.1 Materials

The PLLA fillers employed in this investigation, PLLA‐b‐PEG microsphere (180 mg/mL) and hyaluronate (17 mg/mL); (cross‐linked sodium hyaluronate gel with PLLA‐b‐PEG microspheres), were synthesized by Imeik Technology Development Co. Ltd. (Beijing, China). Enzyme‐linked immunosorbent assay (ELISA) kits were procured from Shanghai Enzyme‐linked Biotechnology Co. Ltd. (Shanghai, China).

### 2.2 Animals

A total of 12 male Japanese white rabbits (4 months of age) were sourced from Beijing Longan Experimental Animal Breeding Center (Beijing, China). Furthermore, 12 male guinea pigs (aged 8 weeks old), 12 male rats (8 weeks old), and 12 male mice (8 weeks old) were obtained from Beijing Changyang Xishan Farm and Beijing HFK Bioscience Co. Ltd. (Beijing, China), respectively. The animals were housed under standardized conditions with a temperature of 23°C ± 3°C and a relative humidity of 40%–70% under a 12‐h light/12‐h dark photoperiod. They were provided with ad libitum access to standard diets and purified water. All animal experiments adhered to the guidelines of the Ethics Committee (IACUA No. YXKT2023L007).

### 2.3 Experimental Process

Following a 1‐week acclimatization period, the animals were anesthetized with 5% pentobarbital sodium or isoflurane (Qingdao Orbiepharm Co. Ltd., Qingdao, Shandong, China), and then simultaneously implanted with the PLLA fillers by injection (0.5 mL) into the periosteum and subcutaneous tissue bilaterally along the spine, adjacent to the plane of the anterior superior iliac spine.

### 2.4 Histological Analysis

Biopsies of the filler and surrounding subcutaneous tissue were performed under pentobarbital sodium anesthesia at 1, 2, 4, and 12 weeks post‐injection. The collected biopsy samples were then fixed with 10% neutral buffer formalin. After decalcification of the bone within the samples, all tissues were embedded in paraffin wax. The samples were sectioned into 4‐μm thick slices using a microtome along the longitudinal axis, stained with hematoxylin and eosin (H&E) and Masson's trichrome stain, and subsequently visualized under a light microscope (BX63, Olympus Corporation, Tokyo, Japan). The inflammatory response surrounding the injection site was scored using ISO 10993 evaluation criteria [24], and the collagen content at each injection site was quantified and analyzed using Image Pro Plus 6.0 (Media Cybernetics Inc. Bethesda, MD, USA).

### 2.5 ELISA

Biopsy samples collected at 1, 2, 4, and 12 weeks post PLLA implantation were flash‐frozen in liquid nitrogen, homogenized, and analyzed according to the manufacturer's instructions for the ELISA kits to determine the expression levels of tumor necrosis factor‐alpha (TNF‐α), interleukin 12 (IL‐12), transforming growth factor‐beta (TGF‐β), and alpha‐smooth muscle actin (α‐SMA), which are commonly utilized to detect inflammatory responses and collagen production associated with implants [10].

### 2.6 Statistical Analysis

Statistical analyses and graph generation were performed using GraphPad Prism 8 software (GraphPad Software, San Diego, CA, USA). Data are presented as mean ± standard error of the mean (SEM). All results were analyzed using Tukey's multiple comparisons test, with statistical significance defined as *p* < 0.05. Descriptions of statistical significance are provided in the figure legends.

## 3 Results

### 3.1 H&E Staining Results After PLLA Filler Injection

H&E staining demonstrated pronounced inflammatory responses across all four animal models at both injection layers, 1 week following PLLA filler injection. In general, the majority of models exhibited a decline in inflammatory response initially, which was followed by a subsequent increase. Variations in the magnitude of the response were observed among varied animals. In the case of mouse subcutaneous tissue, inflammation reached its peak at week 1 and thereafter, decreased and gradually stabilized within the second week. In rat subcutaneous tissue, no significant changes in inflammatory response were noted from weeks 1 to 12. In guinea pig subcutaneous tissue, inflammation began to decrease by week 2; whereas in rabbit subcutaneous tissue, it appeared at week 4. Further, regarding the periosteal injection site in mice, inflammation peaked at week 1, subsequently decreased, and stabilized. In rats, inflammation escalated from weeks 1 to 12. In guinea pigs and rabbits, inflammation of the periosteum reached its peak at week 4, followed by a decline.

Further, a comparison of the different injection layers in the same animals showed similar inflammatory trends in the two injection layers in mice; however, the inflammation levels were consistently higher at the periosteal compared to the subcutaneous tissue at each time point. In rats, the subcutaneous tissue inflammation showed minimal variation over the 12‐week observation period, whereas inflammation of the periosteum increased. Moreover, inflammation levels were elevated at the periosteal layer compared to the subcutaneous tissue in both mouse and rat models. In guinea pigs and rabbits, the subcutaneous tissue and periosteum exhibited contrasting inflammatory response patterns, with the subcutaneous tissue initially showing a decreasing trend followed by an increasing one, whereas the periosteum initially increased and then decreased. Nonetheless, overall inflammation levels in both layers were not significantly disparate (Table 1).

**TABLE 1:** Evaluation of inflammatory.

Layers	Times	Mice	Rats	Guinea pigs	Rabbits
Subcutaneous tissue	1 week	2.7 ± 0.7	4.0 ± 1.2	4.7 ± 0.7	4.7 ± 1.3
	2 week	0.0 ± 0.0	4.0 ± 0.0	2.0 ± 0.0	7.0 ± 3.0
	4 week	0.7 ± 0.7	2.7 ± 0.7	10 ± 1.2	3 ± 3
	12 week	0.7 ± 0.7	4.7 ± 0.7	12 ± 1.6	12 ± 1.2
Periosteum	1 week	4.7 ± 0.7	3.3 ± 0.7	4.0 ± 0.0	4.0 ± 1.2
	2 week	2.7 ± 1.8	3.3 ± 0.7	6.0 ± 0.0	3.3 ± 0.7
	4 week	2.0 ± 1.2	4.7 ± 1.8	9.3 ± 0.7	8.7 ± 2.4
	12 week	2.7 ± 1.2	9.3 ± 1.4	9.0 ± 0.6	7.3 ± 1.3

### 3.2 Molecular Biological Analysis After PLLA Filler Injection

**FIGURE 1:**
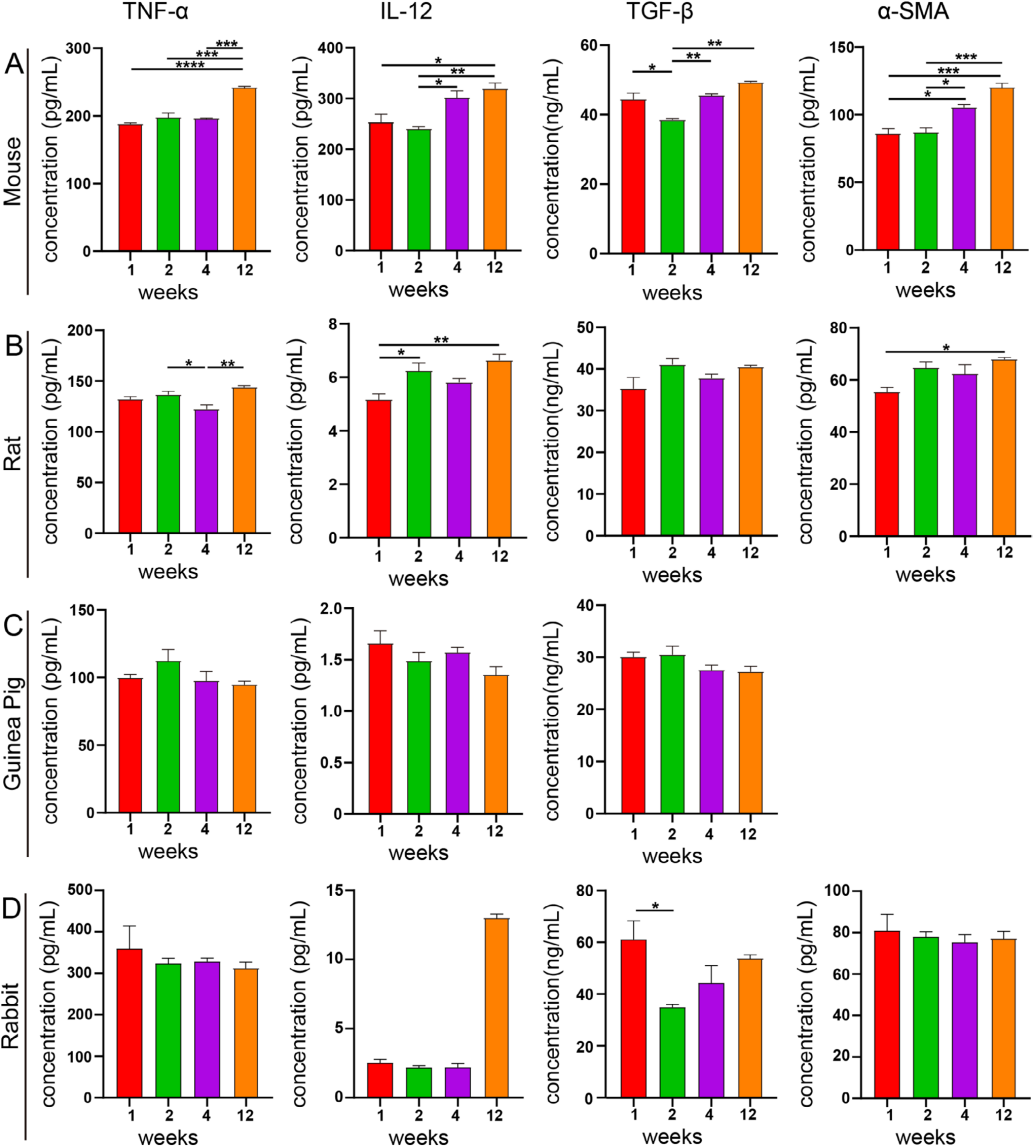
TNF‐α, IL‐12, TGF‐β, and α‐SMA levels in the skin of mice (A), rats (B), guinea pigs (C), and rabbits (D) were measured using ELISA. Data are presented as the mean ± standard error of the mean (SEM) (*n* = 3 per group). *p < 0.05, **p < 0.01, ***p < 0.001, ****p < 0.0001.

ELISA analysis revealed a gradual increase in the expression levels of TNF‐α, IL‐12, TGF‐β, and α‐SMA in mice at 1, 2, 4, and 12 weeks post‐injection (Figure 1A). Comparable findings were observed in rats (Figure 1B). In contrast, the expression levels of TNF‐α, IL‐12, and TGF‐β in guinea pigs exhibited minimal change at various time points following PLLA injection (Figure 1C). Similary, in rabbits, the levels of TNF‐α and α‐SMA remained stable at 1, 2, 4, and 12 weeks post‐injection. In contrast, IL‐12 levels reached their peak at week 12, whereas TGF‐β peaked within the first week post‐injection, followed by a decrease and subsequent rise (Figure 1D).

### 3.3 Analysis of Masson Staining Results After PLLA Filler Injection

The Masson's trichrome staining results demonstrated a progressive increase in collagen content within both the periosteum and subcutaneous tissue of all four animal models over the course of 1 to 12 weeks post‐injection (Table 2). In the mouse and rat subcutaneous tissues, a more rapid rate of collagen regeneration was observed during weeks 1 and 4, which subsequently diminished. In contrast, the guinea pig and rabbit models exhibited rapid collagen deposition from weeks 1 to 2. In the periosteum of mice, the collagen regeneration trend mirrored that observed in the subcutaneous tissue. In rats, sustained collagen production was observed, while in guinea pigs, collagen content only slightly increased. For rabbits, the collagen regeneration pattern in the periosteum was akin to that in the subcutaneous tissue, which rapid regeneration from week 1 to week 2, followed by a decreasing trend.

**TABLE 2:** Evaluation of newly synthesized collagen (%).

Layers	Times	Mice	Rats	Guinea pigs	Rabbits
Subcutaneous tissue	1 week	14.0 ± 2.5	28.9 ± 6.2	35.0 ± 1.8	41.2 ± 1.2
	2 week	9.7 ± 0.2	37.3 ± 4.4	50.1 ± 7.4	55.7 ± 0.3
	4 week	29.9 ± 3.7	44.0 ± 3.0	55.6 ± 1.7	53.1 ± 2.7
	12 week	33.9 ± 1.7	53.7 ± 4.7	61.3 ± 3.7	60.6 ± 1.3
Periosteum	1 week	3.6 ± 0.5	10.7 ± 3.8	32.7 ± 2.0	17.9 ± 4.6
	2 week	8.6 ± 0.4	13.8 ± 3.6	31.2 ± 0.37	45.5 ± 1.4
	4 week	33.8 ± 3.2	26.7 ± 4.3	28.3 ± 2.0	40.0 ± 3.1
	12 week	39.4 ± 3.9	51.0 ± 4.3	47.7 ± 0.9	61.7 ± 2.3

**FIGURE 2:**
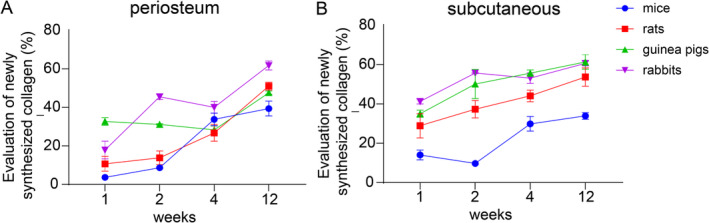
Collagen production in the periosteum (A) and subcutaneous tissue (B) of mice, rats, guinea pigs, and rabbits at various time points post‐PLLA filler implantation is evaluated.

Comparative analyses of collagen production across different injection layers within the same animals yielded similar trends and levels of collagen synthesis in the two layers for mice and rabbits. However, in rabbits, collagen production was more rapid and its peak occurred earlier, within weeks 1 and 2. In rats, collagen production levels were comparable in both layers, but were faster in the periosteum. In guinea pigs, collagen production was slower in the periosteum than in the subcutaneous tissue. Notably, the subcutaneous tissue of rabbits and guinea pigs displayed the most prominent expression of neoformed collagen, followed by that in rats, with less pronounced expression observed in mice. In the periosteum, the expression of nascent collagen was most prominent in rabbits, followed by rats and mice, with guinea pigs showing less expression (Figure 2; and Figures S1–S4).

## 4 Discussion

In the present study, we investigated the inflammatory response in four animal models recommended by ISO 10993‐6 for the assessment of implants [24]. Overall, following the implantation of PLLA in the subcutaneous tissue and periosteum, all four species exhibited significant inflammatory responses and regeneration of collagen between 1 and 12 weeks postimplantation. Notably, the magnitude of the inflammatory response varied among the animals. The level of inflammation was also observed to correlate with animal size, with larger animals presenting elevated inflammation and a pronounced burst of new collagen expression between 1 and 4 weeks, whereas smaller animals showed milder inflammatory responses. Furthermore, the expression levels of TNF‐α, IL‐12, and TGF‐β were upregulated in both rats and mice, but not in guinea pigs and rabbits. Additionally, inconsistencies in inflammatory response were noted between different injection layers within the same animal. In rats and mice, the periosteum exhibited higher inflammation compared to the subcutaneous tissue, whereas guinea pigs and rabbits showed the opposite trend. Further, collagen expression in the subcutaneous tissue was generally greater than in the periosteum.

In recent years, minimally invasive facial rejuvenation techniques have gained widespread popularity because of their low risk, rapid recovery, and significant therapeutic outcomes [25]. Among these techniques, PLLA products, which stimulate collagen regeneration [26, 27], have garnered considerable global attention, and it has been observed that following injection, PLLA microparticles induce mild inflammation [28], which, in turn, effectively promotes collagen synthesis and counteracts the degradation of elastic tissue and collagen associated with facial aging. This process ultimately addresses issues such as skin volume loss and wrinkle formation [16]. Several animal experimental studies [12] have been conducted to explore the mechanism of action of PLLA fillers. Despite utilizing different PLLA fillers and demonstrating similar trends in tissue response and inflammation levels, discrepancies remain regarding the choice of animal models and the layer of implantation. Moreover, inconsistencies persist regarding the selection of animal models for various experimental purposes. In this study, we employed the same PLLA microspheres across different animal models to minimize the influence of material variation on the test outcomes. The findings indicated that all four animal models can serve as nonclinical models for evaluating the safety and efficacy of fillers, as they exhibited comparable inflammatory responses to foreign body stimulation and collagen regeneration. These results align with those reported in previous investigations. Furthermore, ISO 10993 explicitly states that these animals can be utilized to assess tissue reactions to subcutaneous implants [24].

We observed a significant correlation between the tissue inflammatory response and animal body size following subcutaneous PLLA implantation in a study involving four animals. Mice exhibited the lowest level of inflammation, which initially peaked at the implantation onset and then stabilized at a low level, potentially obscuring the implant's inflammatory stimulatory effects. Minimal collagen production was also noted in mice. The results for rats mirrored those of mice, albeit with higher levels of tissue response. Given the widespread use of rodents in scientific studies [29], their significance in in vivo filler evaluation is undeniable. Guinea pigs demonstrated elevated inflammation and collagen production, suggesting their skin's heightened sensitivity to implants, making them more suitable for safety assessment. However, their hypersensitivity to stimulation, along with frequent environmental disturbances, may lead to their demise [30]. Rabbits also exhibited significant tissue response and collagen regeneration. Rabbit ears are often used to study emboli caused by intravascular injection as evidenced by the visibility of blood vessels in the ear [31]. Rabbits can survive for 7–10 year [32], rendering them appropriate for long‐term tissue response evaluation and the assessment of permanent fillers' degradation properties. According to ISO 10993 [24], guinea pigs are suitable for allergy tests, whereas rabbits are better for skin irritation tests. Thus, we propose that rabbits are more suitable for observing long‐term tissue reactions to implanted materials and for evaluating degradation properties and adverse events following intravascular injection, whereas guinea pigs are more appropriate for evaluating implant safety, particularly with regard to sensitization and skin irritation after filler implantation. Consequently, specific model animals may be more suitable for particular research questions.

Furthermore, implantation depth within the same animal also influenced tissue responses [19]. Clinically, deep facial augmentation has been associated with a lower incidence of adverse reactions compared to superficial augmentation [33]. Our study revealed that rabbits and guinea pigs exhibited slightly higher inflammatory responses to PLLA in the subcutaneous tissue than in periosteum, whereas rats and mice showed the opposite trend. This discrepancy may be attributed to the mechanical tension at the implantation site [34]. All four animal models demonstrated similar skin expansion characteristics following the implantation of 0.5 mL of PLLA. Rats and mice, with smaller parietal skin areas, experienced a stronger mechanical stretch upon implantation, resulting in microtrauma and triggering a reparative immune response, thereby increasing inflammation [35]. In contrast, the larger parietal skin area of rabbits and guinea pigs led to lower levels of mechanical tension and inflammation. Larger animal sizes imply larger skin areas and more easily differentiated skin tissue layers, increasing the number of layers and potential injection methods [36]. Although both layers can be used to assess tissue response to fillers, the back appears to be a more suitable implantation site, considering the size of the implanted area and the ease of manipulation during testing. Our findings suggest that all four animals can serve as models for evaluate the effects of deep implants, and implantation into the periosteum is also recommended. Notably, the volume of the implant is a critical factor, as a larger implant volume may elicit unexpected tissue reactions.

Additionally, recent research on PLLA implants has progressively transitioned from the observation of tissue responses to the investigation of underlying mechanisms [12, 37]. To compare the molecular biological differences among various animals following PLLA implantation, the expression of relevant cytokines and proteins postimplantation was assessed using ELISA. Consequently, we noted an increase in levels of tissue inflammation‐related factors, including TNF‐α, IL‐12, TGF‐β, and α‐SMA at the implantation sites in rats and mice. This trend was congruent with histopathological findings. Several studies have confirmed that PLLA stimulates collagen production through foreign body stimulation [10, 38], and the pro‐inflammatory cytokines, pro‐collagen regeneration‐related factors, and associated mechanisms have been extensively studied [10, 38]. Therefore, it has been observed that PLLA induces sustained collagen production by eliciting a mild inflammatory response in tissues. Our experiments demonstrated similar results in rat and mouse models. However, in rabbits and guinea pigs, the expression levels of these pro‐inflammatory cytokines did not undergo significant changes. This observation may be attributed to immunological differences among species and variations in the sensitivity to foreign stimuli, with mice being particularly sensitive [39]. Furthermore, considering the higher antibodies levels in mice, it is plausible that the sensitivity of the immunoassay may vary among different species [40]. Moreover, all the mice used in this study were inbred strains, thereby minimizing the impact of individual differences on the experimental results [41]. Due to the limited availability of molecular biological data from other species, such as the diversity of antibodies and the limitations of analytical methods and techniques, rats and mice are more suitable for analyzing the mechanism of action of implants [42].

In summary, the use of all four animal models in nonclinical studies involving PLLA filler materials facilitates the clear observation of tissue response to implants. Based on our findings, rabbits are more appropriate for evaluating inflammatory response and collagen content, whereas guinea pigs are better suited for safety assessment. Both rats and mice are suitable for studying implant‐related molecular biological indicators, with the back skin being a favorable site for implantation during inflammation response studies. All experiments yielded valid positive results across different animal models. However, from ethical, economic, and animal welfare perspectives, rats appear to be more suitable for noncommercial studies examining tissue responses and the mechanisms of action. In certain types of experiments, animal characteristics, such as body size and lifespan, may not align with the evaluation criteria for fillers. Therefore, for in vivo studies, it is crucial to select appropriate animal models based on the objectives of the clinical evaluation and the characteristics of the animals involved. Collectively, the results of this study may serve as a reference for the nonclinical evaluation of fillers in small animals.

In this study, we compared the physiological responses of various animal species to implantation of PLLA fillers across different tissue layers and drew preliminary conclusions. However, the study had certain limitations. Firstly, the relatively short duration of the animal experiments may have restricted the comprehensive assessment of the long‐term effects of the implants. Secondly, the limitations of molecular assays may not fully capture the complexity of the changes in inflammatory response. A more comprehensive approach is required to detect and analyze these changes, as well as the associated mechanisms. Furthermore, this study solely discusses the evaluation of biodegradable implants using small experimental animal models. Large animals may exhibit responses more akin to humans, and given the variability in responses among different breeds of experimental animals, further investigation in this regard is necessary.

## 5 Conclusion

In this study, we observed comparable trends in the responses of our experimental animal models to PLLA fillers. Our findings revealed that the injection of PLLA filler at the same site in various animals resulted in varying degrees of inflammatory response and collagen production. Furthermore, distinct tissue layers within the same animal exhibited diverse tissue responses following PLLA implantation. We conducted a comprehensive analysis and discussion of the factors contributing to this variability, which may serve as a reference for the nonclinical evaluation of fillers. Additionally, during the nonclinical evaluation, process, it is essential to consider the differences between injection sites and the actual clinical application site to achieve more rational test outcomes. Furthermore, the selection of appropriate animal models must be tailored to the specific scenario under investigation.

## Author Contributions

Chen Lai conceived the idea and designed the experiments. Tong He and Xiaoting Zhang performed the animals experiment. Tong He and Zilin Zhang performed data analysis with the help of Huanyun Niu. Tong He and Zilin Zhang wrote the manuscript with the supervision of Shiwei Wang and Qiaoli Wang. Chen Lai revised the manuscript. All authors approved the final version of the manuscript.

## Ethics Statement

All the animal experiments were conducted in accordance with the guidelines of the Ethics Committee of Beijing YongXin KangTai Technology Development Co. Ltd. and were approved by the aforementioned Ethics Committee (IACUA No. YXKT2023L007).

## Conflicts of Interest

The authors declare no conflicts of interest.

## Supporting information


**Figure S1.** Histological response of mouse periosteum and subcutaneous tissues to PLLA filler injection determined via H&E and Masson’s trichrome staining (scale bar, 50 μm). PLLA, poly‐L‐lactic acid; H&E, hematoxylin and eosin.
**Figure S2.** Histological response of rat periosteum and subcutaneous tissues to PLLA filler injection determined via H&E and Masson’s trichrome staining (scale bar, 50 μm). PLLA, poly‐L‐lactic acid; H&E, hematoxylin and eosin.
**Figure S3.** Histological response of guinea pig periosteum and subcutaneous tissues to PLLA filler injection determined via H&E and Masson’s trichrome staining (scale bar, 50 μm) PLLA, poly‐L‐lactic acid; H&E, hematoxylin and eosin.
**Figure S4.** Histological response of rabbit periosteum and subcutaneous tissues to PLLA filler injection determined via on H&E and Masson’s trichrome staining (scale bar, 50 μm). PLLA, poly‐L‐lactic acid; H&E, hematoxylin and eosin.

## Data Availability

The data that support the findings of this study are available from the corresponding author upon reasonable request.
