# Supplementary material for: Effects of Poly‐L‐Lactic Acid Fillers on Inflammatory Response and Collagen Synthesis in Different Animal Models
Source: J Cosmet Dermatol. 2025 Feb 5;24(2):e70000. doi: 10.1111/jocd.70000 (PMC11799711; doi:10.1111/jocd.70000)
Supplement: Supplementary file 1 — Figure S1. Histological response of mouse periosteum and subcutaneous tissues to PLLA filler injection determined via H&E and Masson’s trichrome staining (scale bar, 50 μm). PLLA, poly‐L‐lactic acid; H&E, hematoxylin and eosin. Figure S2. Histological response of rat periosteum and subcutaneous tissues to PLLA filler injection determined via H&E and Masson’s trichrome staining (scale bar, 50 μm). PLLA, poly‐L‐lactic acid; H&E, hematoxylin and eosin. Figure S3. Histological response of guinea pig periosteum and subcutaneous tissues to PLLA filler injection determined via H&E and Masson’s trichrome staining (scale bar, 50 μm) PLLA, poly‐L‐lactic acid; H&E, hematoxylin and eosin. Figure S4. Histological response of rabbit periosteum and subcutaneous tissues to PLLA filler injection determined via on H&E and Masson’s trichrome staining (scale bar, 50 μm). PLLA, poly‐L‐lactic acid; H&E, hematoxylin and eosin. [file JOCD-24-e70000-s001.zip › Supporting_Information.docx]

## Figure S1

Histological response of mouse periosteum and subcutaneous tissues to PLLA filler injection determined via H&E and Masson’s trichrome staining (scale bar, 50 μm). PLLA, poly-L-lactic acid; H&E, hematoxylin and eosin.

## Figure S2

Histological response of rat periosteum and subcutaneous tissues to PLLA filler injection determined via H&E and Masson’s trichrome staining (scale bar, 50 μm). PLLA, poly-L-lactic acid; H&E, hematoxylin and eosin.

## Figure S3

Histological response of guinea pig periosteum and subcutaneous tissues to PLLA filler injection determined via H&E and Masson’s trichrome staining (scale bar, 50 μm). PLLA, poly-L-lactic acid; H&E, hematoxylin and eosin.

## Figure S4

Histological response of rabbit periosteum and subcutaneous tissues to PLLA filler injection determined via on H&E and Masson’s trichrome staining (scale bar, 50 μm). PLLA, poly-L-lactic acid; H&E, hematoxylin and eosin.
