# Supplementary figures and images for: Effects of Poly‐L‐Lactic Acid Fillers on Inflammatory Response and Collagen Synthesis in Different Animal Models
Source: J Cosmet Dermatol. 2025 Feb 5;24(2):e70000. doi: 10.1111/jocd.70000 (PMC11799711; doi:10.1111/jocd.70000)

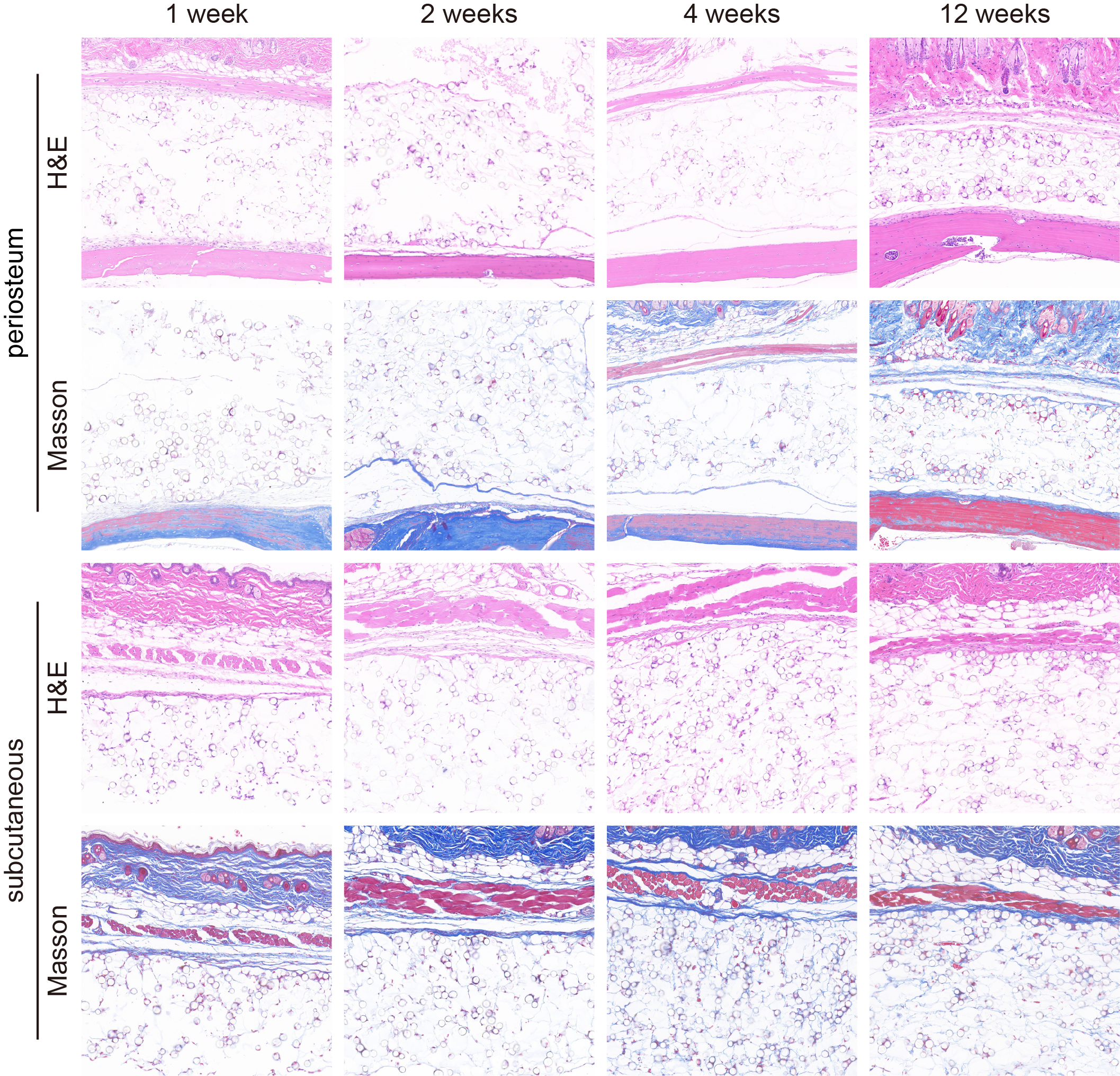

Supplement: Supplementary file 1 — Figure S1. Histological response of mouse periosteum and subcutaneous tissues to PLLA filler injection determined via H&E and Masson’s trichrome staining (scale bar, 50 μm). PLLA, poly‐L‐lactic acid; H&E, hematoxylin and eosin. Figure S2. Histological response of rat periosteum and subcutaneous tissues to PLLA filler injection determined via H&E and Masson’s trichrome staining (scale bar, 50 μm). PLLA, poly‐L‐lactic acid; H&E, hematoxylin and eosin. Figure S3. Histological response of guinea pig periosteum and subcutaneous tissues to PLLA filler injection determined via H&E and Masson’s trichrome staining (scale bar, 50 μm) PLLA, poly‐L‐lactic acid; H&E, hematoxylin and eosin. Figure S4. Histological response of rabbit periosteum and subcutaneous tissues to PLLA filler injection determined via on H&E and Masson’s trichrome staining (scale bar, 50 μm). PLLA, poly‐L‐lactic acid; H&E, hematoxylin and eosin. [file JOCD-24-e70000-s001.zip › Fig_S1.png]

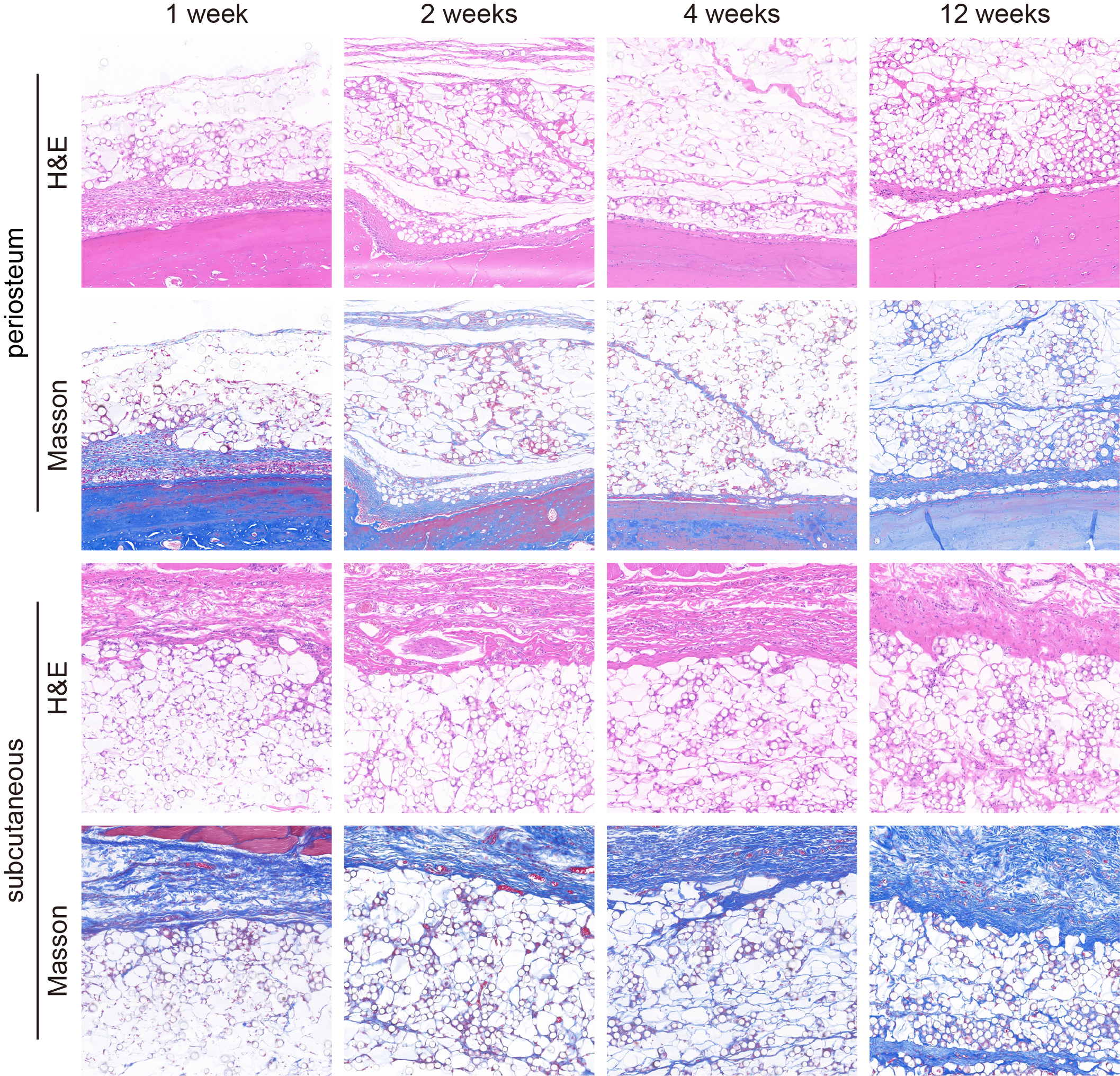

Supplement: Supplementary file 1 — Figure S1. Histological response of mouse periosteum and subcutaneous tissues to PLLA filler injection determined via H&E and Masson’s trichrome staining (scale bar, 50 μm). PLLA, poly‐L‐lactic acid; H&E, hematoxylin and eosin. Figure S2. Histological response of rat periosteum and subcutaneous tissues to PLLA filler injection determined via H&E and Masson’s trichrome staining (scale bar, 50 μm). PLLA, poly‐L‐lactic acid; H&E, hematoxylin and eosin. Figure S3. Histological response of guinea pig periosteum and subcutaneous tissues to PLLA filler injection determined via H&E and Masson’s trichrome staining (scale bar, 50 μm) PLLA, poly‐L‐lactic acid; H&E, hematoxylin and eosin. Figure S4. Histological response of rabbit periosteum and subcutaneous tissues to PLLA filler injection determined via on H&E and Masson’s trichrome staining (scale bar, 50 μm). PLLA, poly‐L‐lactic acid; H&E, hematoxylin and eosin. [file JOCD-24-e70000-s001.zip › Fig_S2.png]

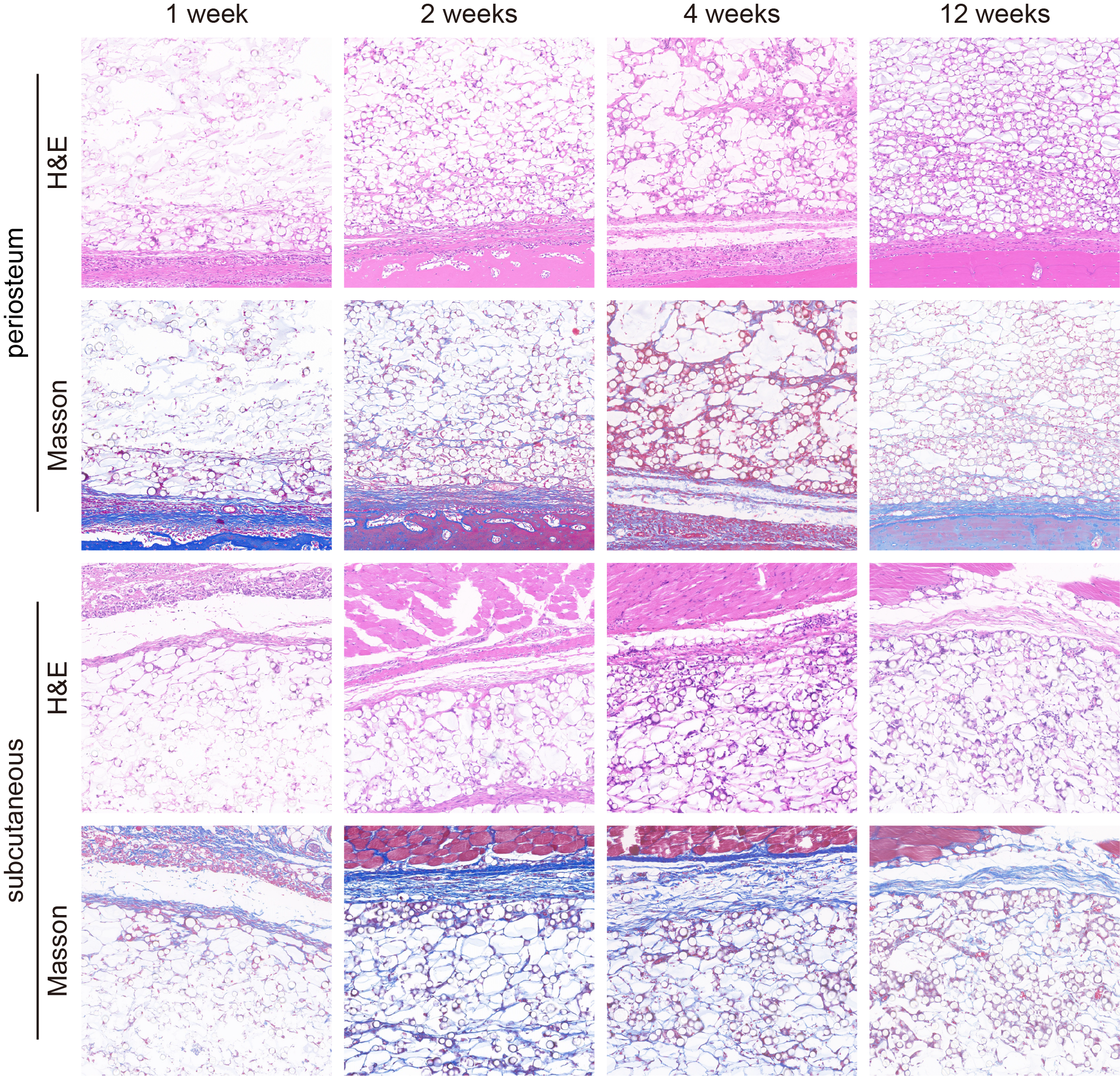

Supplement: Supplementary file 1 — Figure S1. Histological response of mouse periosteum and subcutaneous tissues to PLLA filler injection determined via H&E and Masson’s trichrome staining (scale bar, 50 μm). PLLA, poly‐L‐lactic acid; H&E, hematoxylin and eosin. Figure S2. Histological response of rat periosteum and subcutaneous tissues to PLLA filler injection determined via H&E and Masson’s trichrome staining (scale bar, 50 μm). PLLA, poly‐L‐lactic acid; H&E, hematoxylin and eosin. Figure S3. Histological response of guinea pig periosteum and subcutaneous tissues to PLLA filler injection determined via H&E and Masson’s trichrome staining (scale bar, 50 μm) PLLA, poly‐L‐lactic acid; H&E, hematoxylin and eosin. Figure S4. Histological response of rabbit periosteum and subcutaneous tissues to PLLA filler injection determined via on H&E and Masson’s trichrome staining (scale bar, 50 μm). PLLA, poly‐L‐lactic acid; H&E, hematoxylin and eosin. [file JOCD-24-e70000-s001.zip › Fig_S3.png]

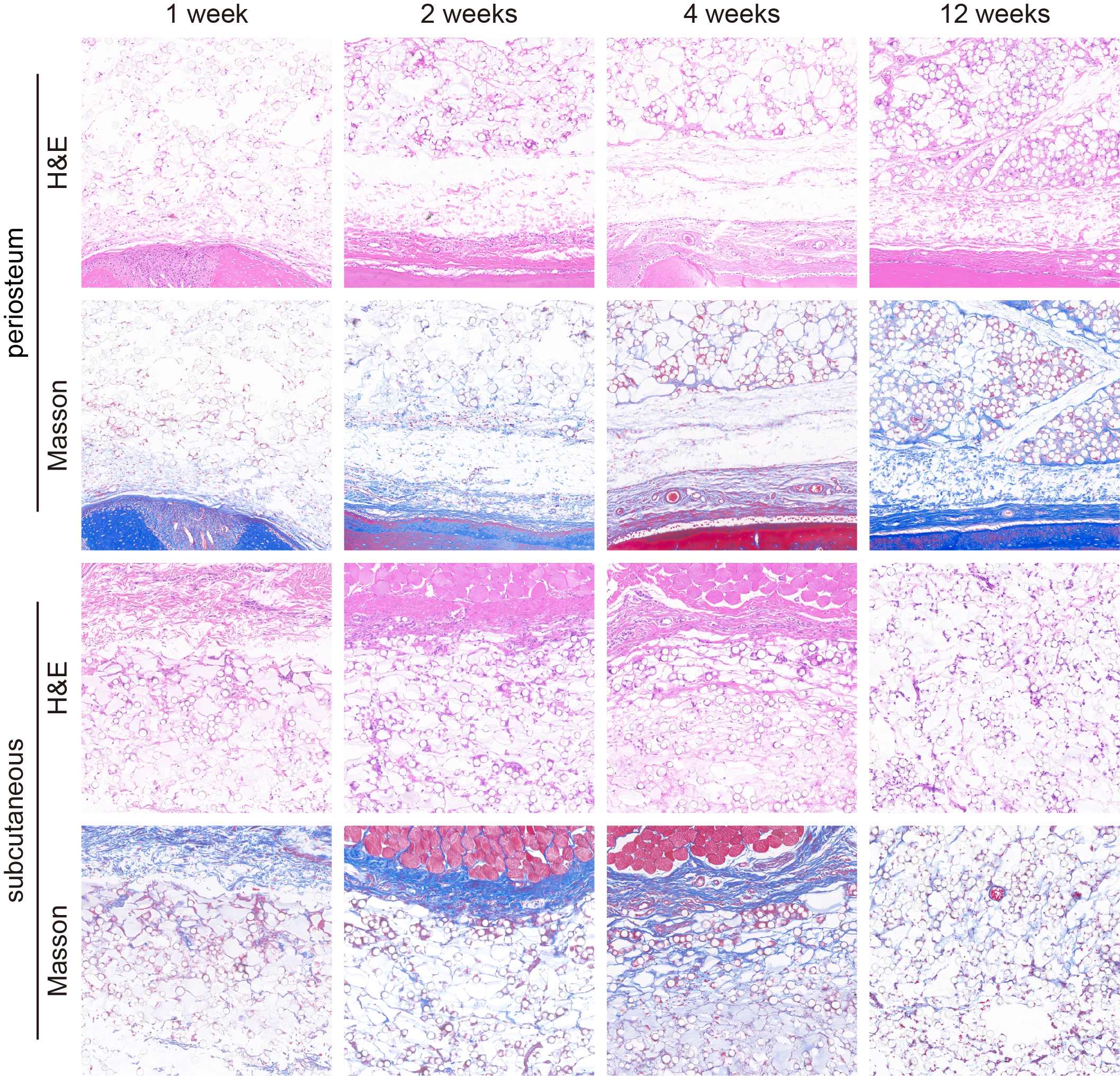

Supplement: Supplementary file 1 — Figure S1. Histological response of mouse periosteum and subcutaneous tissues to PLLA filler injection determined via H&E and Masson’s trichrome staining (scale bar, 50 μm). PLLA, poly‐L‐lactic acid; H&E, hematoxylin and eosin. Figure S2. Histological response of rat periosteum and subcutaneous tissues to PLLA filler injection determined via H&E and Masson’s trichrome staining (scale bar, 50 μm). PLLA, poly‐L‐lactic acid; H&E, hematoxylin and eosin. Figure S3. Histological response of guinea pig periosteum and subcutaneous tissues to PLLA filler injection determined via H&E and Masson’s trichrome staining (scale bar, 50 μm) PLLA, poly‐L‐lactic acid; H&E, hematoxylin and eosin. Figure S4. Histological response of rabbit periosteum and subcutaneous tissues to PLLA filler injection determined via on H&E and Masson’s trichrome staining (scale bar, 50 μm). PLLA, poly‐L‐lactic acid; H&E, hematoxylin and eosin. [file JOCD-24-e70000-s001.zip › Fig_S4.png]
